# Targeting Spore-Forming Bacteria: A Review on the Antimicrobial Potential of Selenium Nanoparticles

**DOI:** 10.3390/foods13244026

**Published:** 2024-12-12

**Authors:** Faraz Ahmed, Dingwu Zhang, Xiaoyang Tang, Pradeep K. Malakar

**Affiliations:** 1 College of Food Science and Technology, Shanghai Ocean University, 999# Hu Cheng Huan Road, Shanghai 201306, China; farazahmedkhan803@gmail.com; 2 International Research Centre for Food and Health, Shanghai Ocean University, 999# Hu Cheng Huan Road, Shanghai 201306, China; 3 Shanghai Kangshi Food Science and Technology Co., Ltd., Shanghai 201103, China

**Keywords:** antibacterial SeNPs, spore inhibition mechanisms, SeNPs’ oxidative stress, SeNPs’ mode of action

## Abstract

Spore-forming bacterial species pose a serious threat to food plants and healthcare facilities that use high-temperature processing and sterilizing techniques to sanitize medical equipment and food items. These severe processing conditions trigger sporulation, which is the process by which spore-forming bacteria, such as those of the *Bacillus* and *Clostridium* species, begin to produce spores, which are extremely resilient entities capable of withstanding adverse environmental circumstances. Additionally, these spores are resistant to a wide range of disinfectants and antibacterial therapies, such as hydrolytic enzymes, radiation, chemicals, and antibiotics. Because of their ability to combat bacteria through several biological pathways, selenium nanoparticles (SeNPs) have emerged as an effective method for either eliminating or preventing the formation of spore-forming bacteria. This review aims to investigate every potential pathway of entry and mechanism by which SeNPs impact bacterial species that produce spores. Additionally, SeNPs’ antibacterial efficacy against several infections is reviewed. To precisely explain the antibacterial mechanism of SeNPs and the various factors that can affect their effectiveness, more research is necessary.

## 1. Introduction

Spore-forming bacteria are a diverse group of microorganisms that produce endospores to survive in unpredictable and extreme environments. Bacterial spores are highly resistant and dormant structures with the potential to exist during nutrient starvation, suboptimum pH levels, and elevated temperatures [1]. The process by which a typical vegetative bacterial cell changes into a dormant spore in reaction to adverse environmental circumstances is known as sporulation, a major contamination problem in food processing and healthcare industries [2,3].

Bacterial spores are major causative agents of foodborne disease [4], food poisoning [5], and food spoilage [6]. The contamination of bacterial spores has been found in a variety of food products including raw milk [7], dehydrated milk [8], canned foods [9], insect-based foods [10], spices and herbs [11], processed foods [12], pasteurized and chilled foods [13], and acidic sauces [14]. The unique structure of bacterial spores contributes to their exceptional dormancy and resistance, making them unsusceptible to ionizing radiations, mechanical disruption, hydrolytic and catalytic enzymes, wet and dry heat, oxidizing and alkylating agents, high vacuum, and desiccation [15]. Similarly, the inactivation methods used to kill or inhibit the growth of bacterial cells including abrasion, predation by bacteriophages, dynamic gas heating, repetitive freezing and thawing, and the use of nonthermal plasma and supercritical fluids are ineffective against inhibiting bacterial spores [16]. Although different inactivation methods also target essential spore structures such as DNA, the inner membrane, and germinating structures, bacterial spores have developed various preventive mechanisms against DNA damage and rapid repair mechanisms through several different methods [17].

However, recent studies have found SeNPs as novel alternatives for traditional antimicrobial and biocidal agents against spore-producing bacteria. Selenium is a trace metal found in living organisms to catalyze the potential of various antioxidant enzymes. SeNPs are small-sized metallic structures that have attracted considerable attention from researchers for their excellent antimicrobial, antioxidant, and therapeutic applications [18,19]. SeNPs provide an inexpensive, easy-to-use, and low-toxicity substitute for antioxidant and antibacterial applications. Many approaches, such as hydrothermal, template, and laser ablation procedures, can be used to synthesize them [20], yet biological synthesis using bacteria, fungi, and plants is more efficient and non-toxic [21]. As a recount of the novel antimicrobial and anti-infection properties of Se, the objective of this review article is to explore the killing or inhibitory mechanisms of spore-forming bacteria with the use of SeNPs to ultimately reduce food contamination. Moreover, the efficacy of Se against various spore formers is also reviewed, while we emphasize a strong need for detailed and focused research in improving the potential of selenium-based nanotechnology to cope with antibiotic or multidrug-resistant bacterial species. Table 1 presents a comparison between traditional food preservation methods and the use of SeNPs in enhancing food safety.

## 2. Mechanism of Action of SeNPs Against Spore-Forming Bacteria

**Figure 1:**
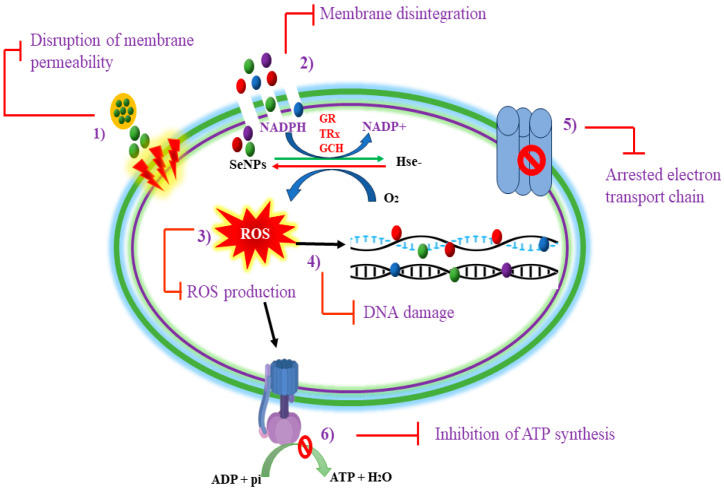
Summary of antibacterial mechanisms of SeNPs.

SeNPs are known to cause cytotoxic effects in bacterial strains through multiple mechanisms including the generation of ROS, the disruption of the cell membrane or inner membrane, the dysfunction of ATP-generating mechanisms, thiol depletion, intracellular penetration, and the release of Se ions [28]. However, the oxidative stress and disruption of cell membranes or penetration of nanoparticles (NPs) within bacteria are well-studied mechanisms elucidating the antibacterial potential of SeNPs in inhibiting the growth of spore-forming bacteria. The antimicrobial mechanisms of SeNPs are illustrated in further detail in Figure 1.

### 2.1. Oxidative Stress

The application of SeNPs increases the production of ROS in bacterial cells, causing irreversible damage to cellular components. ROS are highly reactive molecules with a strong reduction potential, and bacterial cells maintain a balance between ROS and antioxidant mechanisms under normal conditions. While ROS are byproducts of metabolism, their production rises with environmental stressors like antibiotics, heavy metals, or toxins. Bacterial cells possess intrinsic antioxidant defense systems to neutralize ROS; however, exposure to SeNPs overwhelms these defenses. The generation of excess ROS diminishes the activity of key antioxidant enzymes such as superoxide dismutase and catalase [29]. When these mechanisms fail, excessive ROS and free radicals react with biomolecules, leading to metabolic inactivation and reduced cellular viability [30]. SeNPs are known to inhibit the growth of spore-forming bacteria by inducing oxidative stress, once they are absorbed on the surface of targeted bacteria.

In general, SeNPs exhibit photocatalytic properties that enhance ROS generation when exposed to light. This process involves the excitation of electrons in SeNPs, leading to the formation of highly reactive hydroxyl radicals (OH) and singlet oxygen (¹O₂). These ROS are potent oxidants that can damage various cellular components including proteins, lipids, and nucleic acids. The increased production of ROS at the SeNP interface creates a hostile environment for bacterial cells, leading to oxidative stress and subsequent cell death [31].

Wu and Yang [32] investigated the ablation mechanism of selenium-based treatment against both disease-causing Gram-negative and Gram-positive bacterial strains. They found a greater increase in ROS production, especially singlet oxygen molecules, by the application of selenium-containing phenoxazinium than sulfur-based phenoxazinium particles, ultimately inducing cellular stress or apoptosis. Efficient ROS production from SeNPs is related to their simple redox-active mechanism which involves a single-step reduction method to trigger a cycle of oxygen-producing ROS, contributing to its therapeutic potential [33]. Zhang and Li [34] also confirmed the production of ROS from SeNPs as a major mechanism in inhibiting spore-forming bacteria, *Bacillus subtilis,* and cereus strains. In addition to causing oxidative stress, biosynthesized SeNPs also affected the membrane permeability of bacterial cells stimulating the leakage of proteins and polysaccharides outside the cell. Similarly, Sahoo and Panigrahi [31] determined the efficacy of biogenic SeNPs against spore-forming and biofilm-forming bacteria. They observed the concentration-dependent photolytic activity of SeNPs when exposed to spore-producing *Bacillus subtilis*, inducing ROS production, causing the oxidation of bacterial proteins, lipids, DNA, and membrane disruption, and resulting in cell death.

In short, after the induction of ROS by SeNPs, bacterial death can occur through several interconnected mechanisms primarily driven by oxidative stress. This oxidative damage initiates a cascade of events: lipid peroxidation disrupts membrane integrity, resulting in increased permeability and the loss of essential cellular constituents; protein oxidation impairs enzymatic functions and structural integrity, leading to metabolic dysfunction; and DNA damage, characterized by strand breaks and mutations, compromises genetic stability [34]. The accumulation of these damages triggers cellular stress responses including the activation of apoptosis-like pathways in bacteria, where failure to repair oxidative damage leads to programmed cell death. Furthermore, ROS can activate signaling pathways that enhance the expression of stress-response genes while downregulating essential survival genes, further tipping the balance towards cell death [35]. Collectively, these processes illustrate how ROS serve as a pivotal mediator in the lethal effects of SeNPs on bacterial cells, culminating in their demise through a complex approach that disrupts critical cellular functions and structures.

### 2.2. Cell Wall Disruption

To increase their antimicrobial effects, SeNPs penetrate and disrupt cellular membranes. Although the cell walls of both Gram-positive and Gram-negative bacteria are generally negatively charged, they contain positively charged compounds that attract SeNPs through electrostatic interactions. This interaction facilitates the adhesion of SeNPs to the bacterial surface, leading to localized structural alterations including conformational changes in membrane proteins and lipopolysaccharides, increasing permeability and upsetting the electron transport chain. Once attached to the cell surface, SeNPs create transient pores in the outer membrane. This process is thought to occur through a mechanism involving the aggregation of SeNPs on the membrane, which causes mechanical stress and disrupts the integrity of the lipid bilayer [36]. The transient nature of these pores allows for selective permeability changes that enable the influx of additional nanoparticles and the efflux of intracellular components without immediate lysis. Once inside, SeNPs disrupt cellular processes and cause intracellular contents to leak out, which ultimately results in bacterial cell death [37,38]. Tymoshok and Kharchuk [39] controlled the interaction of SeNPs with *B. subtilis* to determine the potential of Se against spore-forming bacterial species. They enriched the nutrient medium of a bacterial culture with optimal concentrations of NPs and analyzed a rapid uptake of Se by bacterial cells. SeNPs created transient porosity in the outer membrane and penetrated the internal compartments of cells without rupturing them. This penetration ability of the SeNPs demonstrated a promising aspect of using selenium-based nanotechnology as effective drug carriers in therapeutic practices. Khiralla and El-Deeb [40] investigated the antibacterial properties of SeNPs against the outgrowth of spore- and biofilm-forming pathogens including *B. cereus*. They found the minimum inhibitory concentration of NPs effective in controlling the growth of spore-forming bacteria, possibly because of the inhibition of biofilm formation.

Chandramohan and Sundar [41] treated spore-forming bacterial cells with SeNPs and found a significant alteration in the structure of the bacterial cell walls and a reduction in the number of treated cells. The SeNPs caused the growth inhibition of the bacterial cells by targeting the cell wall and causing cell wall fragmentation. They also confirmed the attribution of ROS formation as a mechanism involved in the disruption of bacterial membranes. Similarly, Rui and Gu [42] created a bacterial culture that was enriched with Se, which improved the antibacterial activity of SeNPs. There were notable decreases in bacterial growth, spore formation, biofilm development, toxin production, and resistance gene expression when *Clostridium difficile* was exposed to these living particles. The inhibitory mechanisms that resulted in the outflow of intracellular chemicals and cell death included membrane breakdown, modifications to the permeability and integrity of the membrane, and changes to the membrane motive force. SeNPs’ other antibacterial processes are still poorly understood and need more research for wider microbial uses.

### 2.3. Damage to Intracellular Components

The charge-driven attachment of SeNPs stimulates several structural and morphological changes in bacterial membranes, affecting their integrity and permeability potential [41]. SeNPs interact with intracellular elements such as proteins and nucleic acids after entering bacterial cells through compromised cell walls, impairing metabolism and preventing cell division. They can physically bind to vital biological molecules or produce ROS and free radicals, which can chemically change cellular functions. Notably, SeNPs can intercalate with DNA backbones because of their high affinity with phosphates, partially inhibiting transcription and replication sites by blocking access to RNA polymerase and DNA polymerase and potentially causing cell cycle arrest. This disruption not only affects replication but also compromises the repair mechanisms of DNA which increases the likelihood of mutations and genomic instability [43]. Similarly, when SeNPs bind with ribosomal proteins, the aggregation of NPs occupies the active site of enzymes and oxidizes the side chains of amino acids to produce carbonyls which ultimately cause protein dysfunction within a bacterial cell. Additionally, the aggregation of SeNPs around these proteins create steric hindrance to prevent proper substrate binding and catalysis. The oxidative stress generated by SeNPs further exacerbates this dysfunction by oxidizing amino acid side chains, particularly cysteine and methionine residues, leading to the formation of carbonyl compounds that disrupt protein folding and function [44].

SeNPs produce a cascade of ROS, which upsets the oxidation–reduction equilibrium in cells. The overabundance of ROS generated by malfunctioning bacterial defense mechanisms results in oxidative stress, which interacts with nucleic acids and jeopardizes the genomic integrity of resistant forms like spores. ROS can negatively impact spore viability and have been demonstrated to induce a range of DNA lesions, including base changes, single-strand breaks, DNA–protein crosslinks, and pyrimidine dimers [45]. However, SeNP-generated ROS can cause both site-specific and non-specific changes when they interact with cytoplasmic proteins. ROS agglomerate and alter different amino acid residues, causing non-specific alterations that result in protein breakdown or fragmentation. By binding to and chemically altering particular amino acids, ROS cause site-specific changes at the active sites of enzymes, which hinders their catalytic activity [46]. As SeNPs compromise vital intracellular structures, they interfere with metabolic pathways essential for bacterial survival. The disruption of key enzymes involved in energy production diminishes ATP synthesis, leading to energy depletion. This metabolic impairment affects various biosynthetic pathways, including those responsible for synthesizing amino acids and nucleotides, ultimately resulting in cell death due to starvation or the inability to perform essential functions [47].

It has been found that exposure to MNPs tends to induce genetic repair mechanisms in bacterial species, yet higher concentrations of metallic stressors are found to cause irreparable damage to bacterial genetic material [48]. Similarly, MNPs also destabilize the 30S ribosomal unit of bacterial ribosomes, thereby rendering protein translation. Similarly, different types of NPs including Se are effective in the disruption of biofilm formation around bacterial cells, which makes them more susceptible to the interaction of NPs with DNA and proteins, affecting their viability [49].

### 2.4. Inhibition of Adenosine Triphosphate (ATP) Synthesis

The surfaces of spore-forming bacteria, both Gram-positive and Gram-negative, have a net negative charge. Protons from the extracellular environment are pumped into the membrane by the outer wall’s proton motive force during regular metabolism, generating a proton gradient that controls the production of ATP. The energy currency of the cell, ATP, is essential for several metabolic functions, such as cell division, transcription, translation, and protein modification [50]. Under normal conditions, the proton motive force (PMF) continuously maintains a competition between the proton and MNPs for anionic bacterial membrane sites. Thereby, a limited number of NPs can interact with the bacterial membrane which is unable to cause structural deformation or membrane disruption [51]. However, once inside the bacterial cell through strong membrane-bound interactions, MNPs including Se inhibit the synthesis of ATP and thus related energy-requiring metabolic pathways by interfering with this gradient by promoting proton leakage and destabilizing the membrane potential, which reduces the efficiency of ATP generation. Initially, the presence of SeNPs alters the PMF essential for ATP synthesis. The PMF is generated by the active transport of protons across the bacterial membrane, creating a gradient that drives ATP synthase activity [42,52]. In this regard, Huang and Holden [53] combined SeNPs with a short polypeptide to enhance the interaction of NPs with the bacterial wall and found a significant depletion in ATP levels. The inhibition of the ATP synthesis mechanism may have been due to the direct interference of SeNPs with ATP synthase or due to the production of ROS which ultimately disrupted the ATP synthesis machinery within the bacterial cells. Rui and Gu [42] also found that the synergistic effects of SeNPs disrupt the proton motive force within *C. difficile* stains, inhibit the conversion of ADP into ATP, and deform the outer wall, resulting in a large outflow of bacterial DNA and proteins outside the cell, leading to cell death. Table 2 presents a detailed comparison of the antibacterial mechanisms of SeNPs.

## 3. Therapeutic Approaches of SeNPs Against Several Spore-Forming Bacteria Causing Health Issues

**Figure 2:**
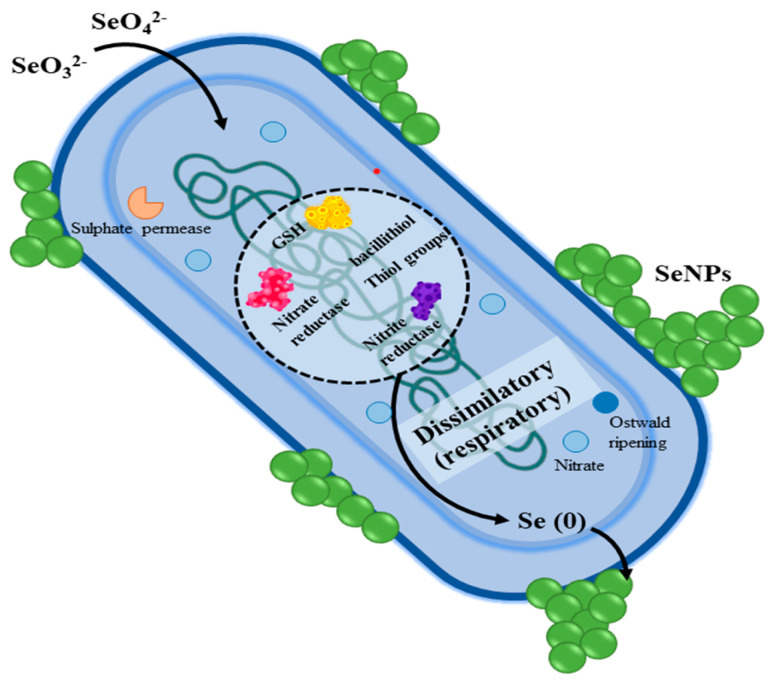
Mechanism of bacterial reduction of selenate/selenite into elemental selenium, Se.

Spore-forming bacteria like *C. difficile, Bacillus anthracis*, *Clostridium botulinum*, *Bacillus cereus*, and *Clostridium tetani* are spore-forming bacteria that can withstand harsh environments by producing robust endospores. These bacteria can cause tetanus, anthrax, colitis, botulism, and foodborne infections. Through oxidative stress, cell membrane damage, spore disruption, and biofilm inhibition, SeNPs suppress these microorganisms. Recent research shows that probiotics containing MNPs and SeNPs have promise in treating bacterial illnesses. Furthermore, environmental bacteria can reduce Se compounds (selenate or selenite) to elemental Se through both respiratory and non-respiratory pathways, as shown in Figure 2. This process significantly lowers the potential environmental impact and reduces toxicity risks to animals. Overall, this mechanism enables bacteria to manage Se toxicity by converting reactive Se compounds into stable elemental forms, thereby enhancing their survival in selenium-rich environments [42,60].

### 3.1. The Antagonistic Effect of SeNP-Loaded Bifidobacterium Breve Against C. difficile

*C. difficile* is a pathogenic bacterium that is frequently found in the human gut. When the balance of gut microbiota is upset, it can lead to serious intestinal infections. This disruption often occurs following antibiotic treatment, which can eliminate beneficial bacteria and allow *C. difficile* to proliferate unchecked. The clinical manifestations of *C. difficile* infections can range from mild diarrhea to severe complications such as pseudomembranous colitis, toxic megacolon, and even death [61].

Recent research indicates that *C. difficile can* enter the human body through various environmental sources, including contaminated water, soil, and food products, like shellfish, and livestock manure. Its resilient endospores contribute to its persistence in these environments, posing a significant risk to public health [62]. Probiotics, especially strains like *Lactobacillus* and *Bifidobacterium*, are gaining attention for their potential to restore gut health and maintain microbial balance in combating *C. difficile* infections. Recognized as safe, they help prevent or mitigate these infections by outcompeting pathogenic bacteria in the gut. Additionally, selenium-enriched probiotics offer a novel functional food approach to enhancing health benefits [63]. Probiotics support gut health by converting inorganic Se into valuable organic forms. This dual action offers a promising strategy to replenish Se levels while providing probiotic benefits. Integrating Se-enriched probiotics into dietary strategies has great potential for preventing and treating *C. difficile* infections by restoring gut microbiota balance, enhancing nutrient absorption, and reducing drug-resistant strains [64,65].

**Figure 3:**
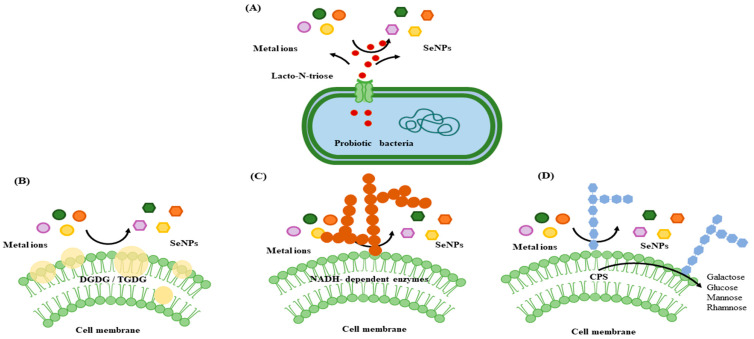
Potential mechanisms for NP synthesis in probiotic bacteria include the following: (**A**) lactose-N-triose-dependent extracellular synthesis, (**B**) lipid-mediated synthesis associated with the bacterial membrane, (**C**) the enzymatic synthesis of NPs, and (**D**) the capsular exopolysaccharide (CPS)-associated reduction in the sugar-mediated synthesis of NPs. In these processes, key components include glycosyl glycosyl diacylglycerol (DGDG), tri glycosyl diacylglycerol (TGDG), and reduced nicotinamide adenine dinucleotide (NADH). CPS plays a significant role in reducing sugars that facilitate NP formation.

To create a strain of *Bifidobacterium* breve that was rich in Se under optimal growth circumstances, single-factor and response surface optimization were used. This strain dramatically reduced *C. difficile ‘s* ability to proliferate, form spores, build biofilms, produce toxins, and express its virulence genes. Probiotic activity and environmental resistance were exceptional in the high-Se B. breve strain. According to the results, *C. difficile ‘s* cell membrane was seriously disrupted, allowing internal components like proteins, ATP, potassium ions, inorganic phosphate, and nucleic acids to leak out. Additionally, cell death was ultimately caused by modifications in the proton motive force (PMF). The mechanisms of cellular toxicity induced by SeNPs are illustrated in Figure 3 [42].

### 3.2. Biogenic SeNPs Against Gram-Positive and Gram-Negative Bacteria

By using bacteria as biological catalysts, biogenic SeNPs can be created, offering a safe and environmentally responsible way to create metal/metalloid NPs with minimal cytotoxicity and high bioactivity without the need for additional stabilizing or reducing agents [66]. Nevertheless, few studies have looked at the antibacterial and antioxidant qualities of biogenic SeNPs and Spirulina. Spirulina and SeNPs are promising substitutes for conventional antibiotics due to their higher bioavailability and lower cytotoxicity, even if antibiotics and chemical antimicrobials successfully stop the growth of pathogens. Future infection prevention may be greatly aided by these innovative agents [67]. In a study by Abdel-Moneim and El-Saadony [18], biogenic SeNPs were produced by Bacillus subtilis AL43, and three Spirulina extracts (methanol, acetone, and hexane) were evaluated for their antibacterial and antioxidant qualities. The findings demonstrated the substantial antibacterial activity of the Spirulina extracts against a range of pathogens and the effective dose-dependent scavenging of DPPH and ABTS radicals, with the methanolic extract showing the highest total phenolic content and superior actions. SeNPs, which are defined as spherical crystalline structures with an average size of 65.23 nm and a negative charge of −22.7 mV, also showed strong antibacterial action against a variety of bacteria and fungi in addition to scavenging radicals. It was shown that the biological activities of the SeNPs and Spirulina were correlated with their total phenolic content. According to these results, the strong antibacterial and antioxidant qualities of Spirulina extracts and SeNPs make them potential candidates for safe and efficient medical uses [18]. The antibacterial effects of SeNPs on different target organisms are summarized in Table 3. The table provides an overview of SeNPs’ characteristics, pathogen-fighting mechanisms, and the observed outcomes of exposure to SeNPs, including the best concentrations for effectiveness and possible host-cell cytotoxicity.

## 4. Sporulation Cycle and Antibacterial Mechanisms of SeNPs

**Figure 4:**
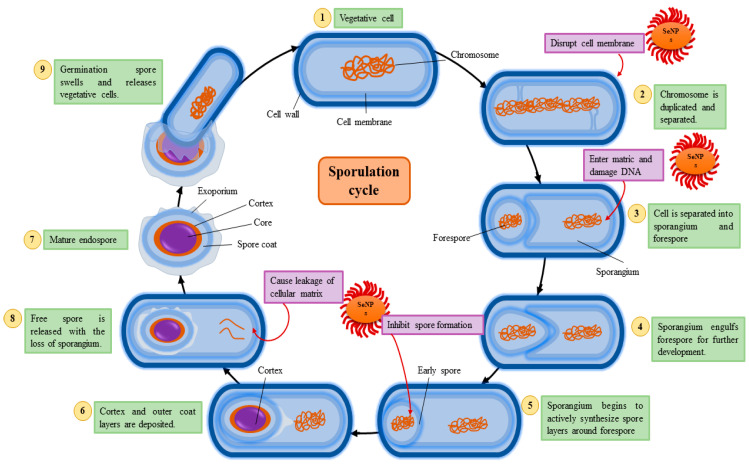
Mechanism of sporulation.

This mechanism demonstrates how SeNPs effectively disrupt bacterial sporulation, a critical survival mechanism in bacteria. Sporulation is a multi-step process where vegetative cells transform into resistant endospores under adverse conditions [81]. This cycle involves DNA duplication, sporangium formation, forespore engulfment, and the deposition of protective layers to produce a mature endospore that can survive extreme environments. SeNPs exert their antibacterial effects in multiple stages of this cycle [82]. They disrupt the bacterial cell membrane, damage DNA during chromosome duplication, and inhibit the synthesis of spore layers, thereby halting spore maturation. Additionally, SeNPs cause the leakage of cellular contents, compromising bacterial integrity and preventing the release of viable spores. By targeting these key processes, SeNPs act as potent antimicrobial agents, offering a promising strategy to control bacterial infections and mitigate spore-related threats [34]. Figure 4 depicts the process of bacterial sporulation, illustrating the transition from a vegetative cell to a mature endospore. The inhibitory effects of SeNPs are highlighted at various stages, including membrane disruption, DNA damage, and the inhibition of spore formation.

## 5. Efficacy of SeNPs Against Spore-Forming Bacteria

High-temperature product processing in food plants and sterilization practices in healthcare fields are traditional methods used to eliminate vegetative cells to prevent sporulation and consequently food spoilage or the spread of infections [83]. However, the prolonged exposure of spore-forming bacteria to high temperatures, pressures, or disinfectant strategies also triggers the expression of spore-forming and resistance genes within vegetative forms. Notably, suboptimal sterilization or processing conditions allow vegetative cells to sporulate or initiate biofilm formation to withstand extreme environmental conditions [84]. MNPs, such as SeNPs, show promise as substitutes for conventional sterilization techniques in lowering the production of spores in food and medication. SeNPs efficiently prevent the growth and sporulation of bacteria, particularly when combined with natural substances or antibiotics [85]. They can more successfully target spore-forming bacteria thanks to this synergy, which strengthens their antibacterial action. Using ascorbic acid to create bimetallic nanomolecules, recent studies have examined the antibacterial potential of SeNPs in combination with platinum nanoparticles [86]. They have exposed bimetallic Pt-Se-NPs to various bacterial species including spore-forming B. cereus cells and found more enhanced bacterial inhibition effects than those achieved using SeNPs alone. Similarly, the use of SeNPs also improves the antibacterial properties of antibiotics against pathogens. Varak and Priya [87] investigated the antimicrobial potential of different antibiotics in combination with SeNPs and found an enhanced synergistic growth inhibition effect with tetracycline against *B. subtilis*. Moreover, the further processing of SeNPs also affects the antibacterial activity of Se against spore-forming bacteria as the surface modification of SeNPs with starch successfully inhibits the growth of *B. cereus* at maximum rates [88]. Zeraatkar and Tahan [89] investigated the synergistic antibacterial effects of biosynthesized SeNPs in combination with conventional antibiotics against multidrug-resistant *P. aeruginosa* and *A. baumannii*. The results showed that SeNPs, even at lower concentrations than antibiotics, effectively inhibited bacterial growth. The checkerboard assay demonstrated a synergistic effect where SeNPs enhanced the antibacterial activity of standard antibiotics, suggesting their potential to overcome antibiotic resistance and improve treatment outcomes in bacterial infections.

SeNPs are being utilized more and more in active food packaging to reduce food spoiling because of their potent antibacterial and antioxidant qualities against spore-forming bacteria [90]. Food preparation can result in bacterial sporulation, which allows dormant spores to survive and germinate in the right circumstances throughout distribution. Numerous processed foods, such as dairy products, foods made from eggs, canned goods, and frozen foods, are contaminated with spore-forming Bacillus and Clostridium species [91]. To reduce potential food contamination possibilities, active food packaging is used to inhibit the growth of microbes within the packaged food [92]. Therefore, the antimicrobial properties of SeNPs against spore formers make them a promising inclusion in active food packaging materials to enhance food quality and reduce the need for the higher level of processing of food products in food plants [93]. Lu and Sameen [94] created a food packaging film using polylactic acid in combination with Se microparticles and found excellent antibacterial and antioxidant activity against food pathogens, with the packaging having better mechanical stability and water-resistant properties. Similarly, Ndwandwe and Malinga [95] improved the microstructure of a nanocomposite starch-based film with SeNPs to investigate the use of Se in edible food packaging. The result showed the enhanced antibacterial potential of the food packaging with the improved tensile strength of the material, suggesting the effective use of SeNPs in biodegradable active food packaging techniques.

The efficacy of antibacterial activities and potential cytotoxicity of SeNPs towards pathogens depend on a variety of factors including the synthesis procedure (physical, chemical, or biological), particle size, dose concentration, duration of exposure, surface charge, molecular weight, and more [96]. As Safaei and Mozaffari [97] confirmed, the maximum activity of SeNPs is dependent on the sodium selenite concentration, level of available nutrients, and incubation period. Another study found the shape and size of SeNPs to be major determinants of antimicrobial activity induced by Se nanostructures where spherical NPs (100 nm) and elongated NPs (1 μm) were investigated [98]. However, when the shape, size, and exposure time of the SeNPs were kept the same, the Se nanostructures demonstrated concentration-dependent antimicrobial activity which was at a maximum at higher concentrations [99]. Similarly, Gunti and Dass [100] determined the concentration-dependent antimicrobial potential of SeNPs through the mechanism of the membrane disruption of spore-forming bacterial and fungal forms. Sun and Shi [101] also found a significantly increased antibacterial activity of biosynthesized SeNPs when used at three times the minimum inhibitory concentration of nanoparticles. They also found the maximum disruption of bacterial membranes at higher doses of SeNPs, which indicated clear bacterial inhibition zones in treated cells.

**Figure 5:**
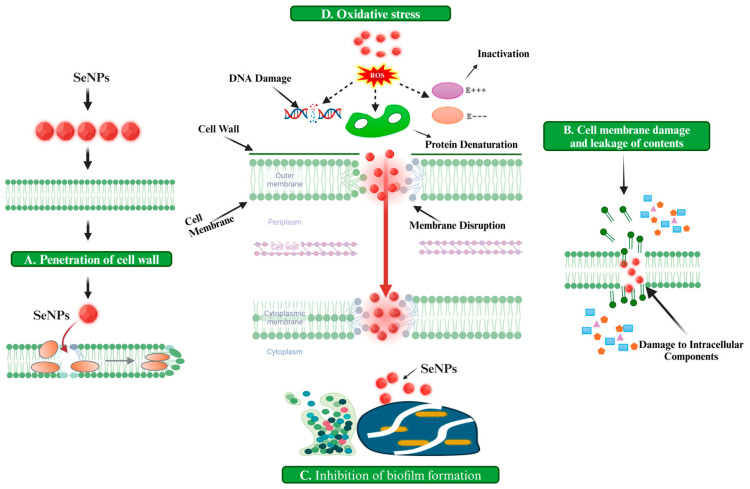
The antibacterial mechanisms of bio-SeNPs emphasize their capacity to enter the bacterial cell wall, damage the cell membrane, allow intracellular contents to seep out, prevent the formation of biofilms, and cause oxidative stress.

The antibacterial potential of SeNPs also depends on the particle size where minimal-sized structures have excellent efficacy against pathogens or disease-causing bacteria, possibly because of the greater surface area available to act [102]. Huang and Holden [50] investigated the size-dependent antimicrobial activity of SeNPs against resistant bacterial species, and particles with an 81 nm diameter showed excellent growth inhibition and killing effects in bacterial cells through different mechanisms such as ATP depletion, ROS production, and membrane deformation. Ref. [103] used biologically synthesized SeNPs with a uniform shape and minimal-size particles against Gram-negative, Gram-positive, and multidrug-resistant bacterial cells to confirm their antibacterial effects. They found that the application of 50–150 nm-sized SeNPs showed the maximum inhibition of bacterial growth because of the higher ROS production and greater potential to penetrate cellular membranes. Figure 5 presents a diagrammatic representation of the antimicrobial and antibiofilm mechanisms of action of SeNPs.

## 6. Synthesis of SeNPs and Their Impact on Antimicrobial Efficacy

SeNPs are synthesized using a variety of methods, each of which significantly impact their antimicrobial efficacy, particularly against spore-forming bacteria. The synthesis methods, broadly categorized into physical, chemical, and biological approaches, directly influence the physicochemical properties of SeNPs including their size, morphology, surface charge, and functionalization [104]. These properties are critical in determining their interactions with microbial membranes, bioavailability, and subsequent antimicrobial activity, which are of paramount importance in food science applications where control over bacterial spores is crucial.

### 6.1. Chemical Synthesis of SeNPs

Chemical synthesis methods such as chemical reduction and hydrothermal techniques offer precise control over the size and uniformity of SeNPs. For instance, chemical reduction involves the use of sodium selenite or selenate as precursors, reduced in the presence of stabilizing agents like polyvinyl alcohol or bovine serum albumin. The chemical reduction method ensures the production of SeNPs with a high level of purity and stability, which translates into consistent antimicrobial efficacy [105]. These nanoparticles are capable of generating ROS upon contact with bacterial spores, leading to the oxidative damage of essential macromolecules including DNA, proteins, and lipids. Furthermore, chemically synthesized SeNPs exhibit high stability in food matrices, ensuring prolonged efficacy during storage and processing [106]. Sentkowska and Pyrzyńska [107] investigated the synthesis of SeNPs via the chemical reduction of sodium selenite using cysteine and ascorbic acid. The synthesis yielded SeNPs with varying sizes and morphologies, influenced by the presence of stabilizers like polyvinyl alcohol and the cleaning techniques applied post-synthesis. Notably, while smaller SeNPs generally exhibited enhanced antioxidant capacities, the homogeneity of the particles also played a critical role. For instance, SeNPs synthesized with a cysteine-to-selenium ratio of 1:4 demonstrated optimal characteristics, as excessive cysteine led to aggregation, negatively impacting antioxidant potential.

However, the chemical synthesis approach has limitations. The use of toxic reducing agents and stabilizers raises concerns about residual contamination in food applications. Additionally, chemical synthesis often involves higher production costs and complex purification steps to ensure that the SeNPs are free from harmful by-products [108]. Despite these challenges, the high degree of control over nanoparticle properties achieved through chemical synthesis methods is unmatched, making it a preferred choice for applications requiring precision such as targeted antimicrobial interventions in food packaging.

### 6.2. Physical Synthesis of SeNPs

Physical methods including laser ablation and thermal evaporation produce SeNPs with a high degree of structural integrity and minimal contamination. For example, laser ablation involves the irradiation of a selenium target submerged in a liquid medium, resulting in the production of SeNPs with a tunable size and high level of stability. These nanoparticles demonstrate enhanced antimicrobial properties due to their uniform size and increased surface area, which facilitate effective interactions with bacterial spores [109]. Physical synthesis methods are advantageous in that they do not require chemical reagents, minimizing the risk of toxic residues in food applications. Haro-Poniatowski and Escobar-Alarcón [110] detailed the synthesis of SeNPs through femtosecond-pulsed laser ablation in liquids using a Coherent-Vitara laser with 24 fs pulse duration and varying energy levels. The SeNPs were generated by ablating a high-purity selenium target submerged in different solvents with ablation times ranging from 1 to 45 min. Characterization techniques including transmission electron microscopy, UV-Vis, and Raman spectroscopy confirmed the formation of nanoparticles sized between 5 and 200 nm with the solvent influencing their crystallinity and morphology, leading to both crystalline and amorphous structures. This synthesis method significantly affected the antibacterial potential of the SeNPs, as evidenced by their ability to inhibit biofilm formation in pathogens like *Candida albicans*, where the particle size and crystallinity were critical factors for efficacy.

Despite their benefits, physical methods are often limited by a high level of energy requirements and low production yields, making them less practical for large-scale applications. Moreover, the equipment and technical expertise required for physical synthesis can increase production costs [111]. However, the superior quality and reproducibility of SeNPs produced by physical methods make them suitable for specialized applications such as antimicrobial coatings for high-value food products.

### 6.3. Biological Synthesis of SeNPs

Biological synthesis methods utilize microorganisms, plant extracts, or enzymatic systems to produce SeNPs. These approaches are gaining attention due to their eco-friendliness and ability to yield biocompatible SeNPs. The resulting SeNPs often possess bioactive coatings of proteins and polysaccharides, enhancing their interaction with bacterial spores. These biologically synthesized nanoparticles exhibit a high level of antimicrobial efficacy due to their ability to disrupt spore germination and outgrowth processes [112]. Ullah and Yin [76] focused on the biosynthesis of SeNPs using the probiotic *B. subtilis* BSN313 where the bacterium effectively reduced selenium concentrations from 5 to 200 µg/mL with a noted decline in the production capability beyond this range. Characterization techniques such as SEM-EDS confirmed the presence of selenium in the nanoparticles which exhibited an average size of 530 nm and a zeta potential of −26.9 mV. Importantly, SeNPs displayed significant antibacterial activity against *E. coli*, *S. aureus*, and *P. aeruginosa* at a concentration of 200 µg/mL alongside good antioxidant properties indicated by DPPH and ABTS scavenging assays.

However, the biological synthesis of SeNPs faces challenges in scalability and consistency. The variability in biological systems often results in SeNPs with heterogeneous size and shape distributions, potentially affecting their antimicrobial efficacy [113]. Despite this, advances in biotechnological methods, such as the optimization of microbial growth conditions and the genetic engineering of synthesis pathways, have shown promise in addressing these limitations.

In short, the choice of synthesis method profoundly affects the antimicrobial potential of SeNPs, particularly in the context of food science. Chemically synthesized SeNPs, with their uniformity and stability, are highly effective in combating spore-forming bacteria like *C. botulinum* and *B. cereus* which pose significant risks in food spoilage and safety [114]. Biological synthesis methods yield SeNPs with biofunctionalized surfaces, enhancing their biocompatibility and reducing cytotoxicity risks in food applications. Physical methods, while less scalable, produce high-purity SeNPs with strong antimicrobial properties suitable for high-precision applications [115].

## 7. Applications of SeNPs in Food Preservation

### 7.1. Use of SeNP in Food Packaging

SeNPs are increasingly incorporated into food packaging materials due to their dual functionality as antimicrobial and antioxidant agents. When embedded in polymers such as polyethene or biodegradable materials like chitosan, SeNPs actively inhibit the growth of bacteria, molds, spores, and other spoilage organisms. This property not only enhances food safety but also significantly extends shelf life [116]. These antioxidant capabilities of SeNPs prevent the oxidative degradation of food and preserve its nutritional and sensory qualities. These packaging systems are particularly relevant for high-risk foods including perishable products where microbial contamination or lipid oxidation is a major concern [117].

In the real world, the incorporation of SeNPs into plastic films and edible coatings is applied directly to food surfaces. These materials actively maintain a protective barrier, ensuring a continuous antimicrobial effect during storage and transportation. Research has shown that foods packaged with SeNP-infused materials exhibit longer shelf life and better preservation of texture, color, and taste compared to traditional methods [118]. For instance, Lu and Sameen [94] investigated the incorporation of SeNPs into PLA films for food packaging applications. Using a solution casting method, various concentrations of SeNPs were added to PLA films, with an optimal performance observed at a 1.5 wt% concentration. The resulting PLA/SeNPs films exhibited enhanced water resistance, UV resistance, and significant antibacterial activity against foodborne pathogens including clostridium species. Although the addition of SeNPs slightly reduced the tensile strength and elongation at the break, the overall properties indicated that these composite films were suitable for food packaging. Recently, Vera and Canellas [119] demonstrated the efficacy of SeNPs incorporated into a flexible multilayer packaging material designed to enhance food preservation. Laboratory tests showed that hazelnuts, walnuts, and potato chips packaged in Se bags exhibited significantly reduced oxidation, with hazelnuts releasing 20% less malonaldehyde, walnuts 25% less, and potato chips 22% less compared to control packaging. An industrial application further confirmed these findings, as cooked ham, chicken, and a vegetable mixture showed over a 25% improvement in shelf life when packaged with antioxidant material. The study highlighted that while the SeNPs did not enhance barrier properties against hexanal and aldehydes, they acted effectively as free radical scavengers.

### 7.2. Direct Food Additive Applications

SeNPs can be utilized directly as food additives to enhance preservation, particularly in processed foods. Their antimicrobial properties are effective against a broad spectrum of foodborne pathogens, reducing microbial loads and spoilage [120]. For example, incorporating SeNPs into liquid foods, dairy products, or baked goods has been demonstrated to suppress bacterial and fungal growth during storage. These nanoparticles act as controlled-release agents to provide prolonged protective effects without altering the flavor or texture [121]. Additionally, SeNPs serve as natural antioxidant additives to delay oxidative spoilage such as lipid peroxidation in oil-based products or discoloration in fruits and vegetables [122]. For the EFSA Panel on Food Contact Materials, Aids [123] evaluated the use of sodium selenite, a coated, granulated preparation of selenium, as a safe and effective food additive in animal nutrition. The findings indicated that, when used within the maximum authorized selenium content in the feed, it posed no significant health risks to consumers or environmental concerns related to soil and water. Overall, sodium selenite is recognized as an efficacious source of selenium for all animal species, contributing to their nutritional needs without raising safety issues.

## 8. Applications of SeNPs in Healthcare

### 8.1. Anticancer Applications

SeNPs have gained attention as potential anticancer agents due to their selective cytotoxicity toward cancer cells while sparing healthy tissues. This selectivity arises from the higher oxidative stress and metabolic activity in cancer cells which makes them more susceptible to the ROS generated by SeNPs [124]. Studies have demonstrated the efficacy of SeNPs in inhibiting tumor growth and inducing apoptosis in cancers such as breast, liver, prostate, and lung cancers. Moreover, SeNPs can be functionalized with targeting ligands to enhance their ability to specifically bind to tumor cells and improve therapeutic outcomes [125]. El-Fakharany and Abu-Serie [126] investigated a novel nanocombination of bovine lactoferrin and SeNPs designed to selectively target invasive cancer cells while sparing normal tissues. The SeNPs were biosynthesized using *Rhodotorula sp.* and characterized to confirm their uniform size (18–40 nm). The resulting ALF-SeNPs demonstrated significant anti-proliferative effects against various cancer cell lines including MCF-7, HepG-2, and Caco-2 with a selectivity index greater than 64 at an IC50 of 63.10 μg/mL. Mechanistically, the ALF-SeNPs upregulated pro-apoptotic genes like p53 while downregulating oncogenes such as Bcl-2 and MMP-9.

### 8.2. Drug Delivery Systems

SeNPs are being increasingly explored as nanocarriers for targeted drug delivery due to their biocompatibility and ability to enhance the bioavailability of drugs. They can be engineered to encapsulate therapeutic molecules ensuring controlled and sustained release at the site of action [127]. The functionalization of SeNPs with targeting ligands such as antibodies or peptides enhances their specificity, reducing off-target effects and improving therapeutic outcomes [128]. Gharbavi and Johari [129] created a novel drug delivery system utilizing niosomes coated with bovine serum albumin and hybridized with SeNPs, resulting in the formulation of NISM-B@SeNPs. The cytotoxicity of the NISM-B@SeNPs was assessed against the A549 cancer cell line, demonstrating significant cytotoxic effects, with notable changes in the expression of apoptosis-related genes (Bax/Bcl-2 ratio) and a non-significant decrease in the expression of the multidrug resistance gene MDR-1. Additionally, biocompatibility studies indicated good safety profiles for the formulation, suggesting that this innovative system holds promise for effective drug delivery and co-delivery applications in biomedicine.

## 9. Environmental Factors Affecting SeNP Activity

SeNPs exhibit unique antimicrobial properties that are influenced by environmental factors such as pH, temperature, and interactions with food matrix components which significantly impact their real-world efficacy against spore-forming bacteria. Among these, pH plays a pivotal role by modulating the surface charge of SeNPs and affecting their interaction with bacterial cell membranes [37]. At an acidic pH, SeNPs exhibit an enhanced antimicrobial efficacy due to the protonation of bacterial membrane components which increases electrostatic attraction between the negatively charged NPs and the positively charged membrane. Conversely, alkaline conditions reduce the efficacy of SeNPs by diminishing this electrostatic interaction, potentially affecting their ability to penetrate bacterial spores or vegetative cells [130]. Menon and Agarwal [131] experimented with pH during the synthesis of SeNPs and demonstrated that varying pH levels significantly influenced the particle size and distribution of SeNPs, with optimal conditions leading to a reduction in size and an increase in the surface area-to-volume ratio. This optimization is directly correlated with NPs’ ability to generate ROS, which plays a pivotal role in their antimicrobial mechanism.

Temperature is another critical environmental factor that modulates the physicochemical properties of SeNPs and their interaction with spore-forming bacteria. Elevated temperatures increase the kinetic energy of SeNPs, enhancing their diffusion and likelihood of interacting with bacterial membranes [132]. Heat stress on bacterial cells also sensitizes them to SeNP-induced oxidative damage by destabilizing membrane lipids and proteins. This combination of thermal stress and NP activity amplifies cell death. On the other hand, at lower temperatures, the reduced kinetic energy and slower ROS generation of SeNPs lead to diminished efficacy [80].

Interactions with other food components also profoundly influence SeNP activity. Proteins present in food matrices can adsorb onto the surface of SeNPs, forming a corona that alters their surface charge and bioavailability. For instance, negatively charged proteins reduce the electrostatic attraction between SeNPs and bacterial membranes, potentially reducing efficacy [93]. In contrast, positively charged proteins facilitate SeNP attachment to bacterial cells, enhancing their antimicrobial properties. Lipids in food matrices also modulate SeNP activity by either sequestering NPs within lipid layers, thereby reducing their availability to interact with bacterial cells, or by serving as additional targets for ROS, indirectly amplifying SeNP-induced damage [133]. Similarly, carbohydrates in food matrices influence SeNP stability; polysaccharides stabilize SeNPs, prolonging their activity, while monosaccharides could reduce ROS generation by acting as competing substrates for oxidative reactions. However, Ndwandwe and Malinga [93] showed that the interaction of SeNPs with food constituents remained below the established safety threshold, indicating no potential toxicity. The study found that the overall migration of starch-based film constituents into food simulants was significantly influenced by the presence of SeNPs, with control films exhibiting higher migration levels across all simulants. Specifically, hydrophilic simulants facilitated greater migration compared to lipophilic ones, attributed to differences in solubility and polarity. Despite these interactions, the migration levels of Se were significantly below the acceptable limit of 60 mg kg−1 set by European regulations. In real-world applications, the complexity of these environmental factors underscores the necessity for a detailed understanding of SeNP behavior under specific conditions [123]. Investigating these factors at a molecular level can lead to tailored applications of SeNPs, ensuring consistent and effective antimicrobial performance in diverse environmental conditions.

## 10. Biocompatibility of SeNPs for Human Cells

The potential toxicity of SeNPs on human cells and beneficial bacteria represents a critical consideration for their application, particularly in the food industry. Despite their established antimicrobial efficacy, SeNPs exhibit varying degrees of cytotoxicity that are influenced by factors such as particle size, dose, surface charge, and functionalization [134]. The cytotoxicity of SeNPs is primarily attributed to their ability to generate ROS. ROS production is a double-edged sword; while it is central to the antimicrobial activity of SeNPs against spore-forming bacteria, excessive ROS levels can also lead to oxidative stress in human cells. Studies using human cell lines, such as HEK293 (human embryonic kidney cells) and HaCaT (human keratinocytes), have demonstrated that SeNPs exhibit dose-dependent toxicity, with higher concentrations inducing significant reductions in cell viability [135]. Thereby, the surface functionalization of SeNPs plays a crucial role in modulating their toxicity. Functionalizing agents such as biocompatible polymers, proteins, or polysaccharides can reduce the adverse effects of SeNPs by stabilizing their structure and preventing uncontrolled ROS generation. For example, SeNPs coated with chitosan or polyethylene glycol have shown reduced cytotoxicity in vitro while maintaining their antimicrobial efficacy [136]. Such surface modifications also influence the biodistribution and clearance of SeNPs, reducing their accumulation in non-target tissues and mitigating systemic toxicity.

The impact of SeNPs on beneficial gut microbiota is another area of concern, particularly for food science applications. Beneficial bacteria such as *Lactobacillus* and *Bifidobacterium* species play a vital role in maintaining gut health and are sensitive to changes in the microenvironment caused by external agents [137]. SeNPs, when ingested, interact with these microbial populations. Their antimicrobial activity is not inherently selective, and excessive exposure to SeNPs can disrupt the balance of gut microbiota, potentially leading to dysbiosis. Dysbiosis is associated with various health issues, including gastrointestinal disorders, weakened immune responses, and metabolic dysfunctions [138]. Research has shown that low doses of SeNPs, particularly those synthesized using green methods, exert minimal effects on beneficial bacteria compared to pathogenic strains, suggesting that precise dosing and synthesis methods are critical in preserving microbial balance [38]. However, the long-term effects of SeNPs on human health remain an area of active investigation. In vivo studies in animal models have provided insights into the biodistribution, metabolism, and excretion of SeNPs. Efforts to enhance the biocompatibility of SeNPs have focused on optimizing their physicochemical properties and tailoring their interactions with biological systems [139].

## 11. Limitations and Future Directions

Although the antibacterial potential of SeNPs is a promising technique for controlling spore-producing bacteria, several constraints limit the use of selenium to control bacterial infections that need to be addressed before their widespread use can be considered. One of the primary concerns is the potential for microbial resistance to SeNPs. Prolonged exposure to sublethal concentrations could drive the evolution of resistant strains, as is often observed with conventional antibiotics and other antimicrobial agents. Understanding the mechanisms by which spore-forming bacteria might adapt to selenium-induced oxidative stress or modify their defense systems is crucial. Exploring strategies to prevent resistance development such as combining SeNPs with other antimicrobial compounds or adopting rotational usage strategies could prolong their efficacy. The longevity of SeNPs’ antimicrobial effects especially in applications like food storage is another factor of consideration where extended protection against bacterial contamination is essential. The stability and sustained release of active selenium species require detailed investigation to ensure prolonged efficacy without compromising food safety or quality. Surface modifications, encapsulation techniques, or integration into smart packaging materials could provide controlled release mechanisms, ensuring consistent antimicrobial activity over time. Studies are needed to evaluate these approaches under various conditions including temperature and humidity variations to establish their practicality in real-world scenarios. The potential environmental impact of selenium nanoparticles also raises significant concerns. Their accumulation in soil and water systems could disrupt ecological balances and harm non-target organisms including beneficial microbes. Research into biodegradable or eco-friendly forms of SeNPs as well as systems to monitor and manage their environmental footprint is critical. Developing SeNPs that degrade into harmless byproducts or ensuring their retrieval after application can mitigate ecological risks. Despite these challenges, SeNPs hold tremendous potential for addressing bacterial infections, but optimizing their design for specific spore-forming bacterial species remains an underexplored area. Spore-forming pathogens such as *Bacillus* and *Clostridium* species vary significantly in their resistance mechanisms, necessitating tailored approaches for maximum efficacy. The surface functionalization of SeNPs with ligands that specifically target spore-forming bacteria can enhance selectivity and minimize unintended effects on human cells or beneficial bacteria. Functionalizing SeNPs with antibodies, antimicrobial peptides, or other bio-recognition molecules could significantly advance their targeted delivery capabilities.

The optimization of SeNP dosage and delivery methods remains another crucial area for future research. The variability in experimental conditions and concentrations used in current studies makes it challenging to determine a standardized dosage that maximizes efficacy while minimizing harm. Rigorous dose–response studies under clinically and environmentally relevant conditions are necessary to establish safe and effective guidelines for SeNP use. Advancing our understanding of the precise mechanisms by which SeNPs exert their antimicrobial effects is equally important. Current evidence suggests that SeNPs disrupt bacterial cell walls, induce oxidative damage, and interfere with intracellular processes such as ATP synthesis. However, detailed studies are needed to elucidate the molecular pathways involved, particularly in spore-forming bacteria. Investigating how SeNPs interact with bacterial spore coats and core components could uncover new strategies to enhance their activity. Similarly, determining how SeNPs affect sporulation and germination processes could provide valuable insights into their role in preventing the re-emergence of pathogens. In applications like food preservation, SeNP integration into packaging materials holds significant promise. Functional packaging that releases SeNPs in response to microbial growth could provide a dynamic defense against contamination. However, the compatibility of SeNPs with food materials, their potential to migrate into food products, and their sensory and nutritional impacts must be carefully evaluated to ensure consumer safety.

Lastly, systematic evaluations of the long-term safety profile of SeNPs are essential before their widespread use. Understanding their interactions with human cells and microbiota, particularly at low and repeated exposure levels, will inform guidelines for antibacterial, environmental, and industrial settings. Large-scale studies focusing on the biodistribution, excretion, and potential toxicity of SeNPs in vivo are needed to address these concerns comprehensively. Therefore, further research in the areas of SeNPs development and improvement of their antibacterial properties is needed. A detailed understanding of the exact targeting mechanisms of SeNPs to specifically attack spore-forming species can significantly enhance their antibacterial efficacy and potential to substitute traditional antimicrobial treatments. It involves the investigation of surface modifications and functionalization experiments to improve the selectivity of nanoparticles. Similarly, determining the synergistic effects of SeNPs can also provide insights into the complementary mechanisms involved in inhibiting spore-forming pathogens as combinations with antibiotics, natural antimicrobial compounds, and other metallic nanoparticles can significantly enhance their anti-infection properties. By addressing these key areas, we can advance the development of effective and safe strategies for combating spore-forming bacterial infections. In conclusion, this critical review sheds light on the promising role of SeNPs as a formidable weapon against spore-forming bacteria. By meticulously exploring oxidative stress, cell wall disruption, intracellular component damage, and the inhibition of ATP synthesis, the review underscores the intricacies of SeNP action. This review not only advances our understanding of the mechanisms behind SeNP antimicrobial activity but also encourages further research and exploration in this domain. As we navigate the challenges posed by spore-forming bacteria, SeNPs emerge as a compelling avenue for future investigations and potential applications in antibacterial strategies and ecosystems.

## 12. Conclusions

In conclusion, the antimicrobial efficacy of SeNPs against spore-forming bacteria represents a significant leap in combating persistent microbial threats. This review highlights the multifaceted mechanisms of SeNPs, including ROS production, membrane disruption, and the inhibition of cellular and spore-forming processes. Beyond their established effectiveness, SeNPs offer a template for innovation, emphasizing biocompatibility, eco-friendly synthesis, and enhanced functionality when integrated with other therapeutic agents. While challenges such as optimal dosage, environmental impacts, and specificity remain, the potential of SeNPs extends into food safety, healthcare, and active packaging technologies. Future investigations should prioritize these aspects to unlock broader applications and ensure their safe, sustainable deployment in antimicrobial strategies.

## Figures and Tables

**Table 1:** Summary of traditional food preservation methods versus use of SeNPs.

Aspects	SeNPs	Thermal Processing (e.g., Pasteurization, Sterilization)	Chemical Preservatives (e.g., Sodium Benzoate, Nitrates)	Natural Antimicrobials (e.g., Essential Oils, Bacteriocins)	Ref.
**Mechanism of Action**	Generates ROS and disrupts microbial cell membranes, proteins, and DNA. Effective against spores.	Kills microbes by denaturing proteins and disrupting cellular processes through heat application.	Alters microbial metabolism and inhibits enzymatic activity through chemical interaction.	Disrupts cell membranes and metabolic pathways; depends on active compounds such as phenols and peptides.	[22]
**Target Range**	Broad-spectrum activity, including spore-forming bacteria (*Clostridium botulinum*, *Bacillus cereus*).	Effective against most vegetative bacteria but less effective against spores without high temperatures.	Effective against broad range of bacteria, but spores often require additional treatments.	Limited to specific strains and less effective against spores.	[23]
**Effect on Food Quality**	Minimal effect on sensory and nutritional properties due to low concentrations needed.	Significant nutrient and texture loss due to high temperatures; impacts sensory attributes.	Potentially alters taste and color of food, depending on chemical used and its concentration.	Risk of altering taste and aroma of food; requires optimization to prevent off-flavors.	[24]
**Environmental Impact**	Green synthesis methods reduce environmental impact; some methods risk contamination with by-products.	Energy-intensive and contributes to greenhouse gas emissions in large-scale applications.	Residues can persist in environment, causing potential ecological damage.	Eco-friendly, but large-scale extraction of natural resources can impact ecosystems.	[25]
**Health Considerations**	Potential cytotoxicity and dysbiosis risks require precise dosing and safety assessments.	Generates potentially harmful compounds (e.g., acrylamide) at high temperatures.	Chemical residues in food are linked to health concerns such as allergies and chronic diseases.	Generally safe, but risk of allergies or sensitivities to specific natural compounds.	[26]
**Stability**	High stability in food matrices; retains antimicrobial activity under varying conditions.	Stability depends on temperature control and duration; prolonged processing risks overcooking or underprocessing.	Stability can decrease over time due to chemical degradation or reactions with food components.	Stability varies with exposure to temperature, light, and oxygen; often requires encapsulation for improved use.	[27]

**Table 2:** A comprehensive comparison of the antibacterial mechanisms of SeNPs.

Mechanism	Description	Key Steps	Applications	Ref.
**1. Oxidative** **Stress**	SeNPs induce the production of ROS, leading to cellular damage.	SeNPs generate ROS upon interaction with bacterial membranes. ROS causes the oxidation of lipids, proteins, and DNA. The accumulation of ROS triggers apoptosis-like pathways.	Antibacterial coatings	[54]
**2. Cell Wall Disruption**	SeNPs penetrate and disrupt bacterial cell walls, compromising structural integrity.	Electrostatic interactions facilitate SeNP adhesion. Transient pores form in the membrane. Increased permeability leads to the leakage of intracellular contents.	Targeted drug delivery	[55]
**3. Inhibition of ATP Synthesis**	SeNPs inhibit ATP production by disrupting the proton motive force and interfering with ATP synthase.	SeNPs destabilize the proton gradient across the membrane. Direct interaction with ATP synthase reduces its activity. The induction of ROS damages components of the electron transport chain.	Cancer therapy	[56]
**4. Membrane Integrity Alteration**	SeNPs affect membrane integrity, leading to ion imbalance and the loss of essential metabolites.	Membrane poration allows for uncontrolled ion flow. The loss of critical ions disrupts metabolic processes. The influx of SeNPs exacerbates membrane damage.	Food preservation	[57]
**5. Induction of Genotoxicity**	SeNPs cause DNA damage through oxidative stress and direct interactions with genetic material.	ROS induce strand breaks and mutations in bacterial DNA. Impaired DNA repair mechanisms lead to cell cycle arrest and death. The loss of genetic stability affects survival.	Antiviral applications	[58]
**6. Inhibition of Biofilm Formation**	SeNPs disrupt biofilm development, enhancing susceptibility to antimicrobial agents.	The downregulation of genes involved in biofilm formation. A reduction in biofilm biomass increases the effectiveness of antibiotics against biofilm-associated bacteria.	Medical device coatings	[59]

**Table 3:** An overview of the effects of SeNPs on different pathogens.

Target Organisms	SeNPs Properties	Mechanism of Action	Effects of SeNP Exposure	References
***Leishmania major***	Spherical and amorphous SeNPs (80–220 nm)	DNA fragmentation	Inhibition of growth of cells and proliferation of cutaneous leishmaniasis	[68]
***Aspergillus fumigatus Candida albicans***	Spherical (80–220 nm)	Unknown	Enhanced antifungal activity and fungal growth inhibition	[69]
***Escherichia coli *** ***Pseudomonas aeruginosa *** ***Staphylococcus aureus***	Spherical (100–400 nm)	Oxidative stress through production of reactive oxygen species	Inhibition of bacterial growth and eradication of already-produced biofilms	[70]
**Multidrug-resistant enteric pathogens**	Spherical (approx. 79 nm)	Oxidative stress	Inhibition of bacterial growth and biofilm formation	[71]
**Multidrug-resistant bacteria and pathogenic fungi**	Isotropic and poly-dispersed spheres (55 nm)	Alteration of membrane structure and oxidative stress	Growth inhibition	[72]
**Fourth-instar larvae of mosquito vectors**	Spherical and elongated (46–78 nm)	Denaturation of cellular components	Larvicidal activity against spread of mosquito vectors	[73]
***Echinococcus granulosus***	Amorphous (80–220 nm)	Membrane disruption and	Strong suicidal effects causing pathogenic killing	[74]
***Escherichia coli Staphylococcus aureus***	Spherical (25–40 nm)	Cytotoxicity and alteration in membrane structure	Growth inhibition	[75]
**Multidrug-resistant bacterial species**	Spherical (25–40 nm)	Oxidative stress and abnormal protein synthesis	Bacteriostatic and bactericidal effects	[47]
***Escherichia coli Staphylococcus aureus Pseudomonas aeruginosa***	Amorphous and spherical (530 nm)	Oxidative stress	Growth inhibition	[76]
***Escherichia coli Staphylococcus aureus *** ***Bacillus subtilis***	Spherical (2–15 nm)	Oxidative stress	Inhibition and degradation of bacterial film	[77]
**Gram-positive and Gram-negative bacteria**	Hexagonal (3–50 nm)	Cell wall lysis and DNA unwinding	Growth inhibition and cell death	[78]
***Stenotrophomonas bentonitica LysiniBacillus sphaericus***	Amorphous and spherical (50–90)	Oxidative stress and DNA degradation	Reduced cellular growth and viability	[28]
***Mycobacterium tuberculosis***	Spherical (40–45 nm)	ROS generation	Growth inhibition and cell death	[79]
***Staphylococcus aureus Escherichia coli Klebsiella pneumonia***	Spherical and monodispersed (80 nm)	ATP reduction, ROS production, and membrane disruption	Enhanced cytotoxicity and cellular inhibition	[53]
***Escherichia coli *** ***Pseudomonas aeruginosa Staphylococcus aureus Staphylococcus epidermidis***	Amorphous and crystalline (1–150 nm)	Membrane penetration and ROS generation	Inhibition of bacterial proliferation	[80]

## Data Availability

No new data were created or analyzed in this study. Data sharing is not applicable to this article.

## Abbreviations

Selenium nanoparticles (SeNPs), reactive oxygen species (ROS), adenosine triphosphate (ATP), adenosine diphosphate (ADP), minimum inhibitory concentration (MIC), minimum bactericidal concentration (MBC), silver nanoparticles (Ag NPs), zinc oxide nanoparticles (ZnO NPs), copper nanoparticles (Cu NPs), ferric oxide (Fe₂O₃), iron nanoparticles (FeNPS), and metallic nanoparticles (MNPs).
